# Supplementary material for: Glycemic effect of post-meal walking compared to one prandial insulin injection in type 2 diabetic patients treated with basal insulin: A randomized controlled cross-over study
Source: PLoS One. 2020 Apr 1;15(4):e0230554. doi: 10.1371/journal.pone.0230554 (PMC7112182; doi:10.1371/journal.pone.0230554)
Supplement: S1 Table — (DOCX) [file pone.0230554.s001.docx]

| **Patient** | **Age in ranges (years)** | **Sex** | **BMI**  **(Kg/m^2^)** | **Diabetes duration (years)** | **OHDs** | **Basal**  **insulin type** | **Basal insulin dose (Unit)** | **HbA1c (%)** | **FPG (mg/dl)** |
| --- | --- | --- | --- | --- | --- | --- | --- | --- | --- |
| 1 | 50-55 | F | 29.6 | 5 | SU, DPP4i | Glargine | 10 | 7.33 | 112 |
| 2 | 55-60 | F | 27.2 | 10 | SU, MFM, PIO, DPP4i | Glargine | 14 | 7.95 | 138 |
| 3 | 65-70 | F | 35.4 | 9 | SU, MFM, SGLT2i | Glargine | 18 | 7.54 | 119 |
| 4 | 60-65 | M | 25.2 | 22 | SU, MFM, DPP4i | Glargine | 44 | 7.28 | 181 |
| 5 | 60-65 | M | 25.0 | 10 | DPP4i | Glargine | 38 | 8.66 | 130 |
| 6 | 60-65 | M | 25.2 | 10 | SU, MFM, PIO | NPH | 12 | 8 | 113 |
| 7 | 60-65 | F | 30 | 8 | SU, MFM | NPH | 12 | 7.79 | 121 |
| 8 | 40-45 | M | 45.9 | 1 | SU, MFM, α- glucosidase inhibitor. | Glargine | 16 | 7.35 | 136 |
| 9 | 50-55 | F | 20.6 | 14 | SU, MFM | NPH | 10 | 7.65 | 117 |
| 10 | 55-60 | M | 35.1 | 15 | SU, DPP4i | Glargine | 26 | 8.14 | 115 |
| 11 | 55-60 | F | 27.4 | 8 | SU, DPP4i | NPH | 24 | 8.46 | 170 |
| 12 | 45-50 | F | 31.6 | 1 | SU, MFM, PIO | NPH | 6 | 7.85 | 153 |
| 13 | 60-65 | F | 28.2 | 20 | SU, MFM | Glargine | 20 | 8.48 | 82 |
| 14 | 55-60 | M | 25.9 | 6 | MFM, PIO, DPP4i, SGLT2i | Glargine | 28 | 8.18 | 151 |

F = female, M = Male, OHDs = Oral hypoglycemic drugs, SU = Sulfonylureas, MFM = Metformin, PIO = Pioglitazone
