# Supplementary material for: Glycemic effect of post-meal walking compared to one prandial insulin injection in type 2 diabetic patients treated with basal insulin: A randomized controlled cross-over study
Source: PLoS One. 2020 Apr 1;15(4):e0230554. doi: 10.1371/journal.pone.0230554 (PMC7112182; doi:10.1371/journal.pone.0230554)
Supplement: S2 Table — (DOCX) [file pone.0230554.s002.docx]

|  | **Post-meal walking** ^a^ | **Basal plus** ^a^ | **Difference between group**  **Co-efficient (95% CI)** | ***p*** |
| --- | --- | --- | --- | --- |
| Total steps/day | 8943.8 ± 882.2 | 6968.8 ± 637.6 | -2086.6 (-3416.4, -756.8) | 0.002 |
| Total post-meal steps/day | 1,482.9 (101.5-6,441.0) | 223.0 (0-459.6) | -1,316 (-2,017.4, -614.5) | 0.001 |
| Total calories/day (Kcal)  Carbohydrate (g) | 1,628.6 ± 58.6  213.5 ± 7.1 | 1,481.5 ± 38.6  190.7 ± 5.0 | -150.5 (-252.5, -48.5)  -23.1 (-36.6, -9.6) | 0.004  0.001 |
| **Breakfast**  Post-meal steps  Duration (min)  Total calories (Kcal)  Total carbohydrates (g)  **Lunch**  Post-meal steps  Duration (min)  Total calories (Kcal)  Total carbohydrates (g)  **Dinner**  Post-meal steps  Duration (min)  Total calories (Kcal)  Total carbohydrates (g) | 214.9 (0-1944.1)  3.5 (0-26.79)  471.0 ± 20.8  61.9 ± 2.6  179.2 (0-1293.6)  6.2 (0-17.0)  542.0 ± 24.3  69.6 ± 3.1  799.8 (17.7-4700.1)  16.0 (0.4-51.7)  488.9 ± 23.9  60.7 ± 3.1 | 19.7 (0-189.3)  0.4 (0-4.6)  462.6 ± 18.8  61.1 ± 2.7  35.6 (0- 245.6)  0.7 (0-4.4)  492.2 ± 22.6  62.0 ± 3.1  55.1 (0-354.2)  1.1 (0-6.4)  462.9 ± 16.8  58.2 ± 2.8 | -175.4 (-468.2, 117.4)  -2.8 (-8.0, 2.5)  -8.2 (-53.1, 36.6)  -0.8 (-7.5, 5.9)  -138.0 (-481.7, 205.8)  -5.4 (-10.2, -0.5)  -51.3 (-108.6, 6.1)  -7.7 (-15.6, 0.1)  -718.3 (-1165.4, -271.1)  -14.5 (-22.4, -6.7)  -27.3 (-77.6, 22.9)  -2.6 (-10.0, 4.9) | 0.228  0.285  0.719  0.814  0.415  0.032  0.080  0.054  0.003  0.001  0.287  0.501 |

^a^ Data are expressed as mean + SE or median (range)
