# Supplementary material for: Glycemic effect of post-meal walking compared to one prandial insulin injection in type 2 diabetic patients treated with basal insulin: A randomized controlled cross-over study
Source: PLoS One. 2020 Apr 1;15(4):e0230554. doi: 10.1371/journal.pone.0230554 (PMC7112182; doi:10.1371/journal.pone.0230554)
Supplement: S3 Table — (DOCX) [file pone.0230554.s003.docx]

| **Patient** | **Sequence of intervention** | **Post-meal walking** | | | | | | | **Basal plus** | | | | | |
| --- | --- | --- | --- | --- | --- | --- | --- | --- | --- | --- | --- | --- | --- | --- |
|  |  | **Meal time** | **Basal insulin dose (unit)** | | **Post-meal steps** | | **Plasma glucose (mg/dl)** | | **Meal time** | **Basal insulin dose (unit)** | | **Prandial insulin dose (Unit)** | **Plasma glucose**  **(mg/dl)** | |
|  |  |  | **wk.0** | **wk.6** | **Number** | **Duration (min)** | **Before meal** | **After meal** |  | **wk.0** | **wk.6** |  | **Before meal** | **After meal** |
| 1 | W→BP | D | 10 | 10 | 1,622  + 295 | 26.2 + 3.7 | 149+25 | 248+22 | D | 10 | 10 | 3.3+0.2 | 166+23 | 139+31 |
| 2 | BP→W | D | 10 | 10 | 1,702  + 162 | 22.5+4.3 | 125+17 | 141+4 | D | 14 | 14 | 4 | 147+11 | 157+18 |
| 3 | W→BP | L or D | 18 | 18 | 1,944  + 171 | 24+ 8.2 | 89+9 | 168+8 | L | 16 | 14 | 4.5+0.2 | 95+5 | 171+16 |
| 4 | W→BP | B or D | 44 | 44 | 2,402  + 374 | 37.5+4.3 | 191+12 | 180+29 | B | 44 | 44 | 4 | 137+6 | 158+13 |
| 5 | W→BP | B or D | 38 | 30 | 1,580  + 285 | 30.0+5.4 | 116+5 | 143+12 | B | 30 | 30 | 3 | 150+10 | 201+17 |
| 6 | W→BP | D | 12 | 8 | 2,674  + 214 | 33.7+3.7 | 202+27 | 206+40 | B | 8 | 8 | 4 | 146+11 | 161+23 |
| 7 | W→BP | ND | 12 | 12 | ND | ND | 142+5 | 209+6 | B | 12 | 12 | 4 | 133+6 | 205+18 |
| 8 | BP→W | ND | 16 | 16 | ND | ND | 159+3 | 172+10 | B | 16 | 16 | 6 | 147+3 | 145+9 |
| 9 | BP→W | ND | 6 | 6 | ND | ND | 160+22 | 235+16 | D | 8 | 6 | 3.3+0.2 | 148+30 | 177+19 |
| 10 | BP→W | D | 26 | 26 | 4,749  + 715 | 51.0+7.6 | 98+6 | 148+17 | B | 26 | 26 | 4 | 113+6 | 168+15 |
| 11 | W→BP | B or D | 22 | 22 | 914+144 | 37.5+7.5 | 160+10 | 178+31 | D | 22 | 22 | 5 | 140+24 | 140+10 |
| 12 | BP→W | B or L | 6 | 6 | 1,253  + 100 | 20.0+5.0 | 146+20 | 188+38 | D | 6 | 6 | 4 | 185+7 | 176+30 |
| 13 | W→BP | L or D | 20 | 20 | 2,829  + 61 | 33.7+3.7 | 164+3 | 182+21 | B | 20 | 20 | 4.6+0.2 | 142+7 | 190+12 |
| 14 | BP→W | B or L | 28 | 28 | 3,028  + 552 | 40.0+7.4 | 116+9 | 193+20 | B | 28 | 28 | 4 | 129+71 | 178+19 |

Plasma glucose data were averaged from weekly self-monitoring of blood glucose. Patient number 2, 4-8, 10-12 and 14 were on the same dose of prandial insulin from wk.0 through wk.6

Data are expressed as mean + SE. B = breakfast, L = lunch, D = dinner, W = post-meal walking, BP = basal plus, ND = not done
