# Supplementary figures and images for: Glycemic effect of post-meal walking compared to one prandial insulin injection in type 2 diabetic patients treated with basal insulin: A randomized controlled cross-over study
Source: PLoS One. 2020 Apr 1;15(4):e0230554. doi: 10.1371/journal.pone.0230554 (PMC7112182; doi:10.1371/journal.pone.0230554)

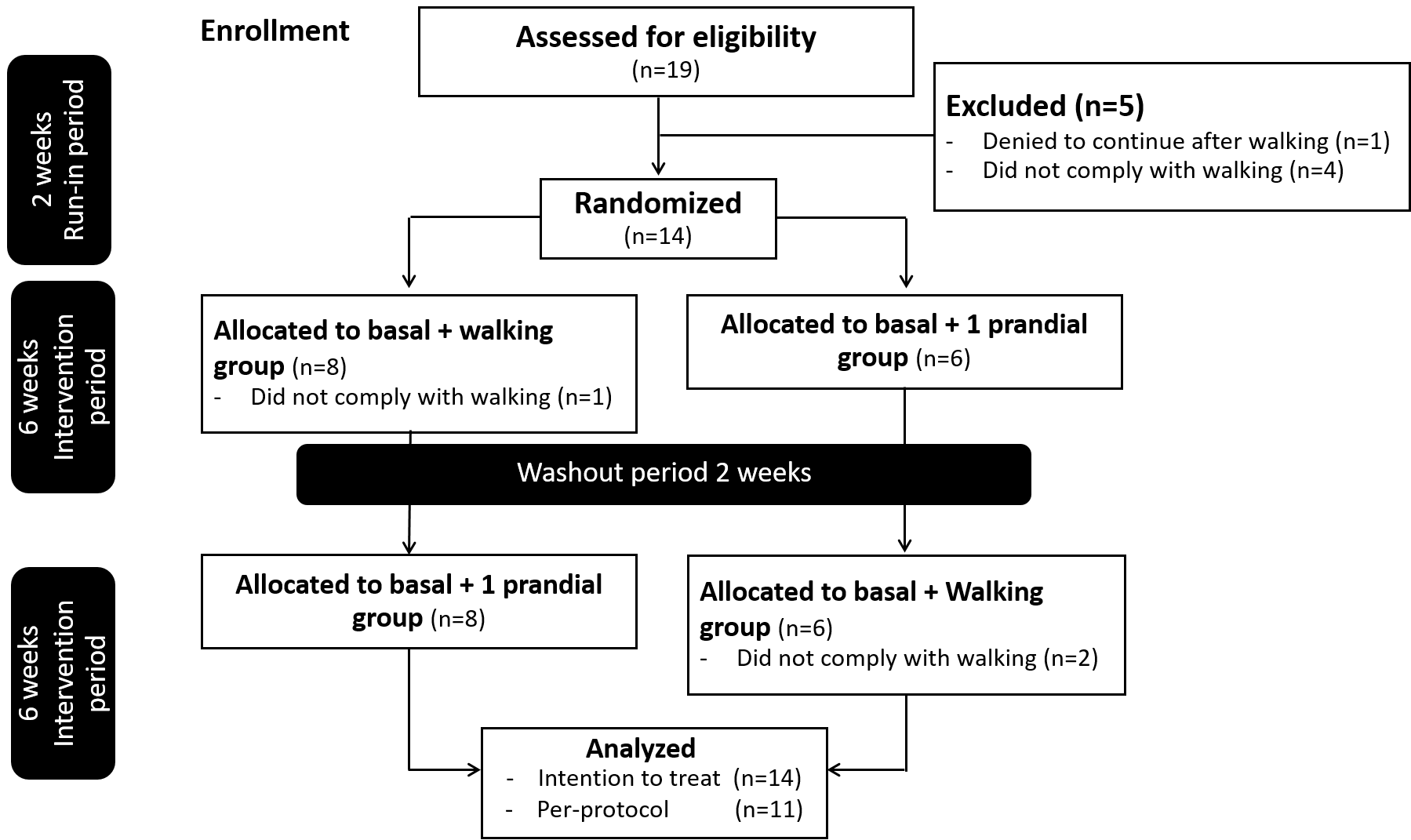

Supplement: S1 Fig — (TIF) [file pone.0230554.s004.tif]

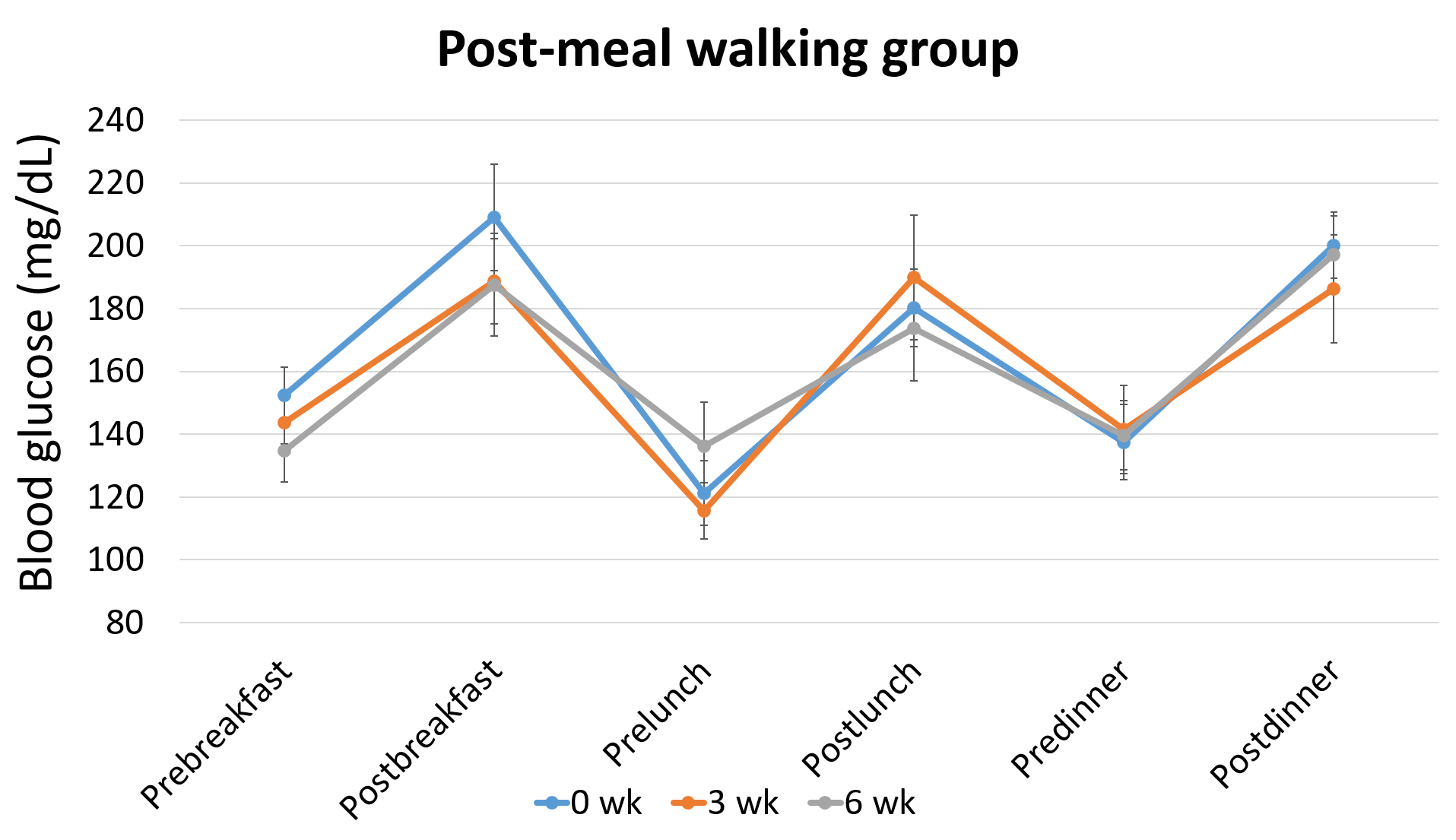

Supplement: S2 Fig — (TIF) [file pone.0230554.s005.tif]

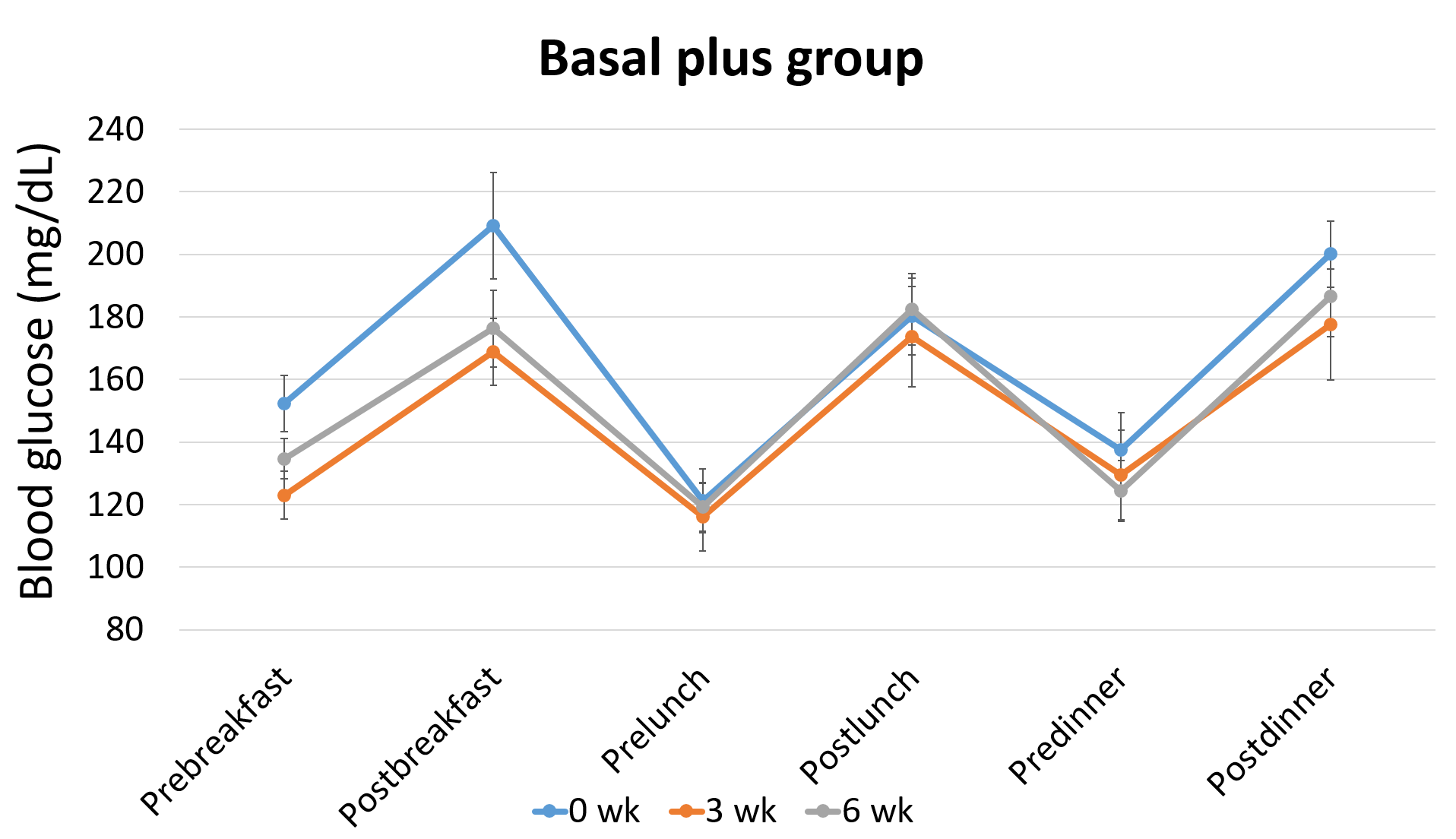

Supplement: S3 Fig — (TIF) [file pone.0230554.s006.tif]
