# Supplementary material for: Glycemic effect of post-meal walking compared to one prandial insulin injection in type 2 diabetic patients treated with basal insulin: A randomized controlled cross-over study
Source: PLoS One. 2020 Apr 1;15(4):e0230554. doi: 10.1371/journal.pone.0230554 (PMC7112182; doi:10.1371/journal.pone.0230554)
Supplement: S1 File — (PDF) [file pone.0230554.s008.pdf]

Research protocol (Full proposal)

Ramathibodi hospital, Mahidol university

1. **Title of the study:** The glycemic effect of post-meal walking with basal insulin compared to basal plus regimen in type 2 diabetes: a Randomized controlled cross-over study

2. **Principle investigator:** Onnicha Suntornlohanakul, M.D.
   Position: Fellow in Endocrinology and Metabolism
   Department of medicine
   Ramathibodi hospital, Mahidol university
   E-mail: Onnicha.sun@gmail.com

3. **List name, affiliation and e-mail address of all sub-investigators and advisor**

Name	Position	Affiliation	E-mail address
Prof.Chatchalit Rattarasarn, M.D.	Advisor	Department of medicine<br>Faculty of medicine<br>Ramathibodi hospital, Mahidol university	Chatchalit.rat@mahidol.ac.th
Associate Prof. Atiporn Ingsathit, M.D, PHD.	Co-investigator	Department of medicine<br>Faculty of medicine<br>Ramathibodi hospital, Mahidol university	Atiporn.ing@mahidol.ac.th
Chatvara Areevut, MS, RD	Co-investigator	Department of medicine<br>Faculty of medicine<br>Ramathibodi hospital, Mahidol university	Chatvara244@yahoo.com
Sunee Saetung, M.Sc.	Co-investigator	Department of medicine	ssaetune@hotmail.com

		Faculty of medicine<br>Ramathibodi hospital, Mahidol university	

4. **Rationale of the study and literature review**

Diabetes is a major public health concern. It can cause diabetic retinopathy, diabetic nephropathy, diabetic neuropathy, cardiovascular disease, cerebrovascular disease and peripheral vascular disease. Patients with diabetes have high morbidity and high mortality.

The prevalence of diabetes increases around the world especially type 2 diabetes. Thai National Health Examination Survey, NHES V found that the prevalence of hyperglycemia in patients older than 15 years old increase from 6.9 to 8.96% (1).

It is well established that good glycemic control with HbA1c 6.5-7% can reduce the microvascular complication and mortality (2-6).

HbA1c is the result of fasting plasma glucose (FPG) and post prandial plasma glucose (PPG). However, FPG and PPG affect HbA1c unequally. Previous study (7) showed that FPG would affect HbA1c more than PPG in patients with high HbA1c levels. For example, patients with HbA1c more than 10.2%, FPG would contribute for 70% of the HbA1c results and PPPG would contribute for 30%. On the other hand, if the patients have lower HbA1c such as 7.3%, FPG would contribute for 30% and PPG would contribute 70%.

Several diabetes guidelines (8) recommend initiation of basal insulin in type 2 diabetic patients after failure to oral hypoglycemic agents. After starting insulin, if FPG is lowered to appropriate ranges but HbA1c has not reached the target, adding medications to reduce the postprandial hyperglycemia is the next step. Adding rapid-acting insulin analog before one main meal (basal-plus) or GLP-1 agonist in combination with basal insulin or switching from basal to pre-mixed insulin are the options. However, with all of these, the patients need more than one injection a day and may increase the risk of hypoglycemia. Moreover, GLP-1 agonist is expensive and has irritable gastrointestinal adverse effects.

Diepietro et al (9) found that in patients who are at risk for diabetes, three bouts of 15 minutes of moderate (3 METs; Metabolic Equivalent Tasks) post-meal walking can significantly improve 24-hour mean plasma glucose. In addition, 15 minutes of post-meal walking was more effective than 45 minutes of sustained walking in lowering of 3-hour post-dinner plasma glucose levels.

Colberg et al (10) showed that post-dinner walking in type 2 diabetic patients decreased PPG at 1 hour after meal about 40 mg/dl compared with no walking.

The study by Van Dijk et al [11] demonstrated that walking after meal for 15 min could reduce PPG in type 2 diabetic patients, but the reduction did not reach statistical significance.

Reynolds et al [12] also demonstrated that glycated albumin was reduced by 10-15 minutes walking after three main meals for two weeks.

From the previous studies, it can be concluded that walking after meal for 10-20 minutes can reduce PPG more effectively than walking before meal and effect of PPG reduction starts since first time of walking. However, most studies are short-term studies and none compares PPG reduction with other standard treatments such as oral hypoglycemic agents or insulin.

Therefore, our study aimed to compare the efficacy of post-meal walking (at least one meal per day everyday) with one prandial insulin on glycemic control in type 2 diabetic patients who failed basal insulin and oral hypoglycemic agents.

5. Objectives of the study

To study the efficacy of post-meal walking compared with prandial insulin in type 2 diabetic patients who failed basal insulin and oral hypoglycemic drugs

Primary outcome: HbA1c reduction at 6 weeks

Secondary outcomes: Fructosamine reduction at 3 and 6 weeks, Self-monitoring blood glucose (SMBG), anthropometric changes, hypoglycemic events, mixed meal test (PPG and TG at 1,2,3 and 4 hours after standardized meal)

Definition of hypoglycemic events

- Glucose alert value (level 1): plasma glucose ≤ 70 mg/dL
- Clinically significant hypoglycemia (level 2): plasma glucose < 54 mg/dL
- Severe hypoglycemia (level 3): neurological symptoms and need assistance

6. Research methodology

Study design: randomized controlled cross-over study

Location of the study/Trial site: Ramathibodi hospital, Bangkok, Thailand

Methods

Run-in period (2 weeks)

1. Recruit the participants and collect the demographic data of participants
   Demographic data
   a. Sex, age, occupation
   b. Information about diabetes
      i. Duration and onset of diabetes
      ii. Complications of diabetes
      iii. Current treatment
      iv. Last HbA1c and FPG

    c. Comorbidities
    d. Anthropometric measurement
2. The participants receive and are instructed how to use glucose meter (Freestyle Optium H, Abbot, USA), and accelerometer (Triaxial accelerometer, Fitbit zip, Fitbit, USA)
3. The participants receive diabetes education which include insulin injection, detection and correction of hypoglycemia, self-monitoring blood glucose (SMBG), and diet.
4. In the first week of run-in period, the participants record the diet in food diary for one day and perform SMBG 6 times a day (before and 2 hours after breakfast, lunch and dinner). The participants must carry the accelerometer during the day to monitor the steps.
5. In the second week of run-in period, the participants revisit clinic to review the food diary and glucose meter use. In this week, participants must walk for 15-20 minutes at least one meal per day every day. The walk should start 15-30 minutes after meal. The participants must carry the accelerometer during the day to monitor the steps. The participants also record the diet and perform SMBG 6 times a day for one day.

At the end of the run-in period, participants are randomized into two groups (post meal walking and basal plus group) by random block design. The blood tests (HbA1c, FPG, serum fructosamine, fasting lipid profile, PPG and TG at 1,2,3,4 hours after standardized meal) are collected before entering each group.

The post meal walking group (Duration: 6 weeks)

1. The participants continue oral hypoglycemic agent and basal insulin as previous.
2. The participants must walk for 15-20 minutes after meal at least one meal per day every day. The speed of walk should be “Walk as fast as possible”. During the day, the accelerometer are used to monitor the number of walking steps.
3. In each week, the participants must record the diet in food diary for one day and perform SMBG 6 times a day (before and 2 hours after breakfast, lunch and dinner).
4. The participants visit the investigator at third week to review the food diary, SMBG and collect the blood tests (FPG, serum fructosamine)
5. The participants visit the investigator at sixth week to review the food diary, SMBG and collect the blood tests (FPG, serum fructosamine, HbA1c, lipid profile, PPG and TG at 1,2,3,4 hours after standardized meal)

The basal plus group (Duration: 6 weeks)

1. The participants continue oral hypoglycemic agent and basal insulin as previous.
2. The participants are advised to use rapid acting insulin analog (Glulisine, Sanofi, France) 15 minutes before the main meal as prandial or bolus insulin. The starting dose are 4 units/meal or 0.1 unit/kg body weight depending on participants’ characteristic.
3. Bolus insulin dose are adjusted as appropriate via electronic devices such as mobile phone or line application within 2 weeks until 2-hour PPG ≤ 180 mg/dl.

4. The participants can do their usual activity but they are advised not to walk after meal.
5. In each week, the participants must record the diet in food diary for one day and perform SMBG 6 times a day (before and 2 hours after breakfast, lunch and dinner).
6. The participants visit the investigator at third week to review the food diary, SMBG and collect the blood tests (FPG, serum fructosamine)
7. The participants visit the investigator at sixth week to review the food diary, SMBG and collect the blood tests (FPG, serum fructosamine, HbA1c, lipid profile, PPPG and TG at 1,2,3,4 hours after standardized meal)

Wash out period

After finishing each intervention group, the participants enter wash out period for 2 weeks. In wash out period, the participants must behave as before participating in the study. At the end of wash-out period, the blood tests (FPG, serum fructosamine, HbA1c, lipid profile, PPG and TG at 1,2,3,4 hours after standardized meal) are collected prior to entering the next intervention group.

Tools and research procedures

1. PPG and TG at 1,2,3,4 hours after standardized meal
   - The test is done in the morning after fasting for 12 hours
   - Plasma glucose and lipid profile are measured at 0 hour
   - The participants take Isocal which contains 256 calories (37% fat, 50% carbohydrates and 13% protein)
   - Plasma glucose and lipid profile are measured at 1,2,3, and 4 hours after that
2. Accelerometer (Triaxial accelerometer, Fitbit zip, Fitbit, USA)
   - This accelerometer has been validated for its accuracy in several trials [13-19].
3. Glucose meter (Freestyle Optium H, Abbot, USA)
4. Blood tests

   - HbA1c: Turbidimetric inhibition immunoassay (TINIA) by Roche
   - Plasma glucose: Hexokinase/Glucose-6-Phosphate Dehydrogenase (HK/G6P-DH) method by Abbot
   - Serum fructosamine: chemiluminescence method by Roche
   - Fasting lipid profile: accelerator selective dependent method by Abbot

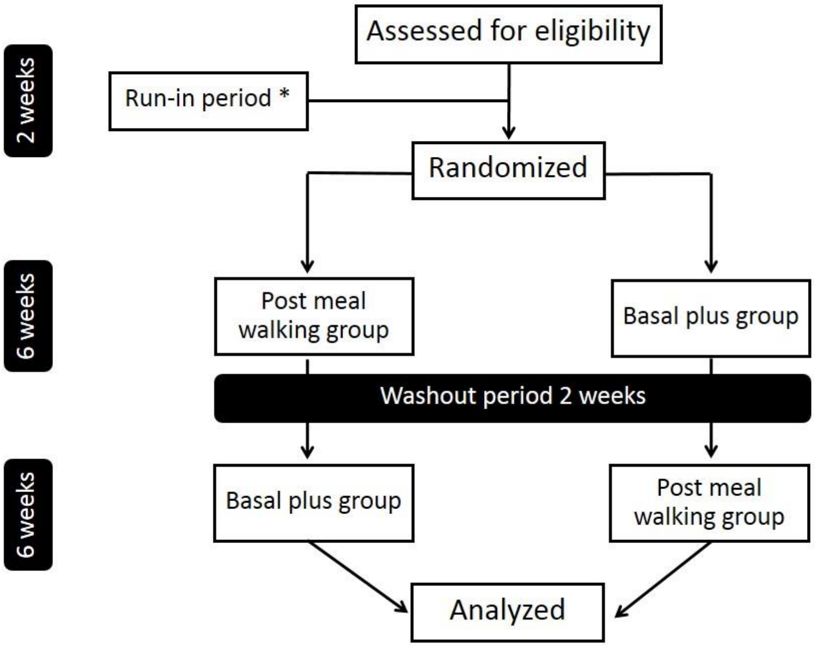

Fig.1 flow of the study

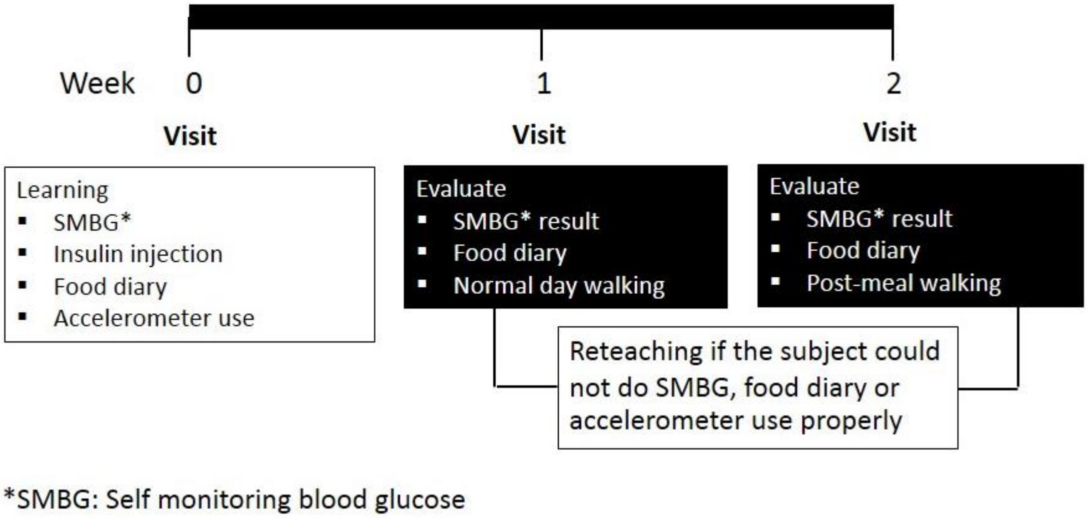

Fig.2 Protocol during run-in period

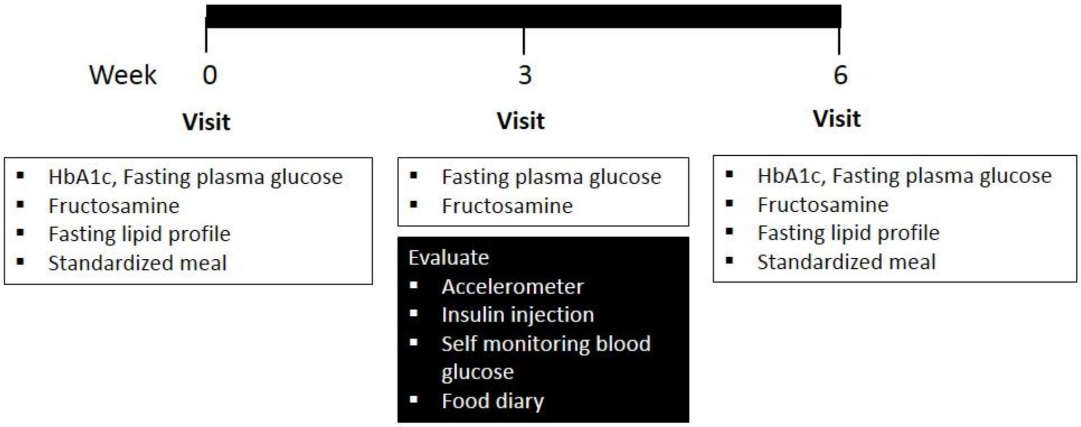

Follow up diagram in both groups

Fig.3 follow up diagram and blood tests in both intervention groups

7. Sample size calculation [20]

$$n = \frac{(z_{1-\frac{\alpha}{2}} + z_{1-\beta})^2 \sigma^2}{\Delta^2}$$

1. σ is standard deviation of the data, according to Lankisch et al [21] the SD of HbA1c is 0.65
2. α (type I error) is 0.05
3. β (type II error) is 0.80
4. Δ (Delta) is the difference of primary outcome between each intervention group. The delta was set at 0.5

At least 14 participants were needed to demonstrate the statistical significance. Assuming there would be 20% of dropout rate, we designed to include 16 participants into the study.

<u>**Inclusion criteria**</u>

1. Type 2 diabetic patients aged 35-70 years with at least one OHD and basal insulin (Intermediate-acting insulin/NPH or Determir or Glargine or Degludec)
2. FPG less than 150 mg/dl and HbA1c levels between 7-9%
3. BMI between 18.5-30 kg/m^2

Exclusion criteria

1. uncontrolled hypertension (systolic blood pressure > 160 or diastolic blood pressure > 100 mmHg)
2. recent myocardial infarction or ischemic stroke within 3 months
3. chronic lung diseases or heart failure
4. foot problems (severe diabetic neuropathy, fracture, deformity, previous amputation) which were obstacle to walking
5. currently took systemic steroids
6. alcohol consumption more than 7 drinks per week or caffeine consumption more than 400 mg/day
7. Travel regularly across time zone or perform shift work

Discontinuation criteria

1. Hyperglycemic emergency (diabetic ketoacidosis, hyperglycemic hyperosmolar state)
2. FPG > 250 mg/dl consecutively or HbA1c > 9%

8. Statistical analysis

The data would be collected by epidata program (ver.3.1) and analyzed by stata program (ver.15)

9. References

1. Aekplakorn W et al 2559;Pageshttp://kb.hsri.or.th/dspace/handle/11228/4604.
2. Intensive blood-glucose control with sulphonylureas or insulin compared with conventional treatment and risk of complications in patients with type 2 diabetes (UKPDS 33). UK Prospective Diabetes Study (UKPDS) Group. Lancet. 1998;352(9131):837-53.
3. Gerstein HC, Miller ME, Byington RP, Goff DC, Jr., Bigger JT, Buse JB, et al. Effects of intensive glucose lowering in type 2 diabetes. N Engl J Med. 2008;358(24):2545-59.
4. Holman RR, Paul SK, Bethel MA, Matthews DR, Neil HA**.** 10-year follow-up of intensive glucose control in type 2 diabetes. N Engl J Med. 2008;359(15):1577-89.
5. Patel A, MacMahon S, Chalmers J, Neal B, Billot L, Woodward M, et al. Intensive blood glucose control and vascular outcomes in patients with type 2 diabetes. N Engl J Med. 2008;358(24):2560-72.
6. Duckworth W, Abraira C, Moritz T, Reda D, Emanuele N, Reaven PD, et al. Glucose control and vascular complications in veterans with type 2 diabetes. N Engl J Med. 2009;360(2):129-39.
7. Riddle M, Umpierrez G, DiGenio A, Zhou R, Rosenstock J**.** Contributions of basal and postprandial hyperglycemia over a wide range of A1C levels before and after treatment intensification in type 2 diabetes. Diabetes Care. 2011;34(12):2508-14.

8. Standards of Medical Care in Diabetes-2017. Diabetes Care. 2017;40(Suppl 1):S1-S135.
9. DiPietro L, Gribok A, Stevens MS, Hamm LF, Rumpler W. Three 15-min bouts of moderate postmeal walking significantly improves 24-h glycemic control in older people at risk for impaired glucose tolerance. Diabetes Care. 2013;36(10):3262-8.
10. Colberg SR, Zarrabi L, Bennington L, Nakave A, Thomas Somma C, Swain DP, et al. Postprandial walking is better for lowering the glycemic effect of dinner than pre-dinner exercise in type 2 diabetic individuals. J Am Med Dir Assoc. 2009;10(6):394-7.
11. van Dijk JW, Venema M, van Mechelen W, Stehouwer CD, Hartgens F, van Loon LJ. Effect of moderate-intensity exercise versus activities of daily living on 24-hour blood glucose homeostasis in male patients with type 2 diabetes. Diabetes Care. 2013;36(11):3448-53.
12. Reynolds AN, Mann JI, Williams S, Venn BJ. Advice to walk after meals is more effective for lowering postprandial glycaemia in type 2 diabetes mellitus than advice that does not specify timing: a randomised crossover study. Diabetologia. 2016;59(12):2572-8.
13. An HS, Jones GC, Kang SK, Welk GJ, Lee JM. How valid are wearable physical activity trackers for measuring steps? Eur J Sport Sci. 2016:1-9.
14. Case MA, Burwick HA, Volpp KG, Patel MS. Accuracy of smartphone applications and wearable devices for tracking physical activity data. Jama. 2015;313(6):625-6.
15. Ferguson T, Rowlands AV, Olds T, Maher C. The validity of consumer-level, activity monitors in healthy adults worn in free-living conditions: a cross-sectional study. Int J Behav Nutr Phys Act. 2015;12:42.
16. Lee JM, Kim Y, Welk GJ. Validity of consumer-based physical activity monitors. Med Sci Sports Exerc. 2014;46(9):1840-8.
17. Schneider M, Chau L. Validation of the Fitbit Zip for monitoring physical activity among free-living adolescents. BMC Res Notes. 2016;9(1):448.
18. Tully MA, McBride C, Heron L, Hunter RF. The validation of Fibit Zip physical activity monitor as a measure of free-living physical activity. BMC Res Notes. 2014;7:952.
19. Kooiman TJ, Dontje ML, Sprenger SR, Krijnen WP, van der Schans CP, de Groot M. Reliability and validity of ten consumer activity trackers. BMC Sports Sci Med Rehabil. 2015;7:24.
20. Chow S-C, Shao,J., Wang, H. Sample Size Calculations in Clinical Research Chapman & Hall/CRC; 2003.
21. Lankisch MR, Ferlinz KC, Leahy JL, Scherbaum WA. Introducing a simplified approach to insulin therapy in type 2 diabetes: a comparison of two single-dose regimens of insulin glulisine plus insulin glargine and oral antidiabetic drugs. Diabetes Obes Metab. 2008;10(12):1178-85.
