# Supplementary material for: Glycemic effect of post-meal walking compared to one prandial insulin injection in type 2 diabetic patients treated with basal insulin: A randomized controlled cross-over study
Source: PLoS One. 2020 Apr 1;15(4):e0230554. doi: 10.1371/journal.pone.0230554 (PMC7112182; doi:10.1371/journal.pone.0230554)
Supplement: S2 File — (PDF) [file pone.0230554.s009.pdf]

แบบเสนอโครงการวิจัยเพื่อรับการพิจารณาจากคณะกรรมการจริยธรรมการวิจัยในคน คณะแพทยศาสตร์โรงพยาบาลรามาธิบดี มหาวิทยาลัยมหิดล (ฉบับเต็ม)

1. **ชื่อโครงการ**

(ภาษาไทย) การศึกษาผลการลดระดับน้ำตาลในเลือดของการเดินหลังอาหารเปรียบเทียบกับการฉีดอินซูลินชนิดออกฤทธิ์สั้นก่อนอาหารมื้อหลัก 1 มื้อ ในผู้ป่วยเบาหวานชนิดที่ 2 ที่ไม่สามารถควบคุมระดับน้ำตาลในเลือดด้วยอินซูลินชนิดพื้นฐาน

(ภาษาอังกฤษ) The glycemic effect of post-meal walking with basal insulin compared to basal plus regimen in type 2 diabetes: a Randomized controlled cross-over study

2. **ชื่อหัวหน้าโครงการวิจัย**

(ภาษาไทย) พ.ญ.อรณิชา สุนทรโลหะนะกูล

(ภาษาอังกฤษ) Onnicha Suntornlohanakul, M.D.

ตำแหน่ง แพทย์ประจำบ้านต่อยอดชั้นปีที่ 1 หน่วยต่อมไร้ท่อและเมแทบอลิซึม

คุณวุฒิ แพทยศาสตรบัณฑิต คณะแพทยศาสตร์จุฬาลงกรณมหาวิทยาลัย
วุฒิบัตรผู้ชำนาญในการประกอบวิชาชีพเวชกรรมสาขาอายุรศาสตร์ (แพทยสภา)

สถานที่ทำงาน สาขาวิชาต่อมไร้ท่อและเมแทบอลิซึม ภาควิชาอายุรศาสตร์
คณะแพทยศาสตร์โรงพยาบาลรามาธิบดี มหาวิทยาลัยมหิดล
e-mail: Onnicha.sun@gmail.com
โทร 087-7164646

3. **ชื่อผู้ร่วมวิจัย**

ผู้ร่วมวิจัย 1 ศ.นพ.ชัชลิต รัตรสาร
Chatchalit Rattarasarn, M.D.

ตำแหน่ง ศาสตราจารย์

คุณวุฒิ แพทยศาสตรบัณฑิต มหาวิทยาลัยสงขลานครินทร์
วุฒิบัตรผู้ชำนาญในการประกอบวิชาชีพเวชกรรมสาขาอายุรศาสตร์ (แพทยสภา)
วุฒิบัตรผู้ชำนาญในการประกอบวิชาชีพเวชกรรมอนุสาขาอายุรศาสตร์
โรคต่อมไร้ท่อและเมแทบอลิซึม (แพทยสภา)

สถานที่ทำงาน สาขาวิชาต่อมไร้ท่อและเมแทบอลิซึม ภาควิชาอายุรศาสตร์

คณะแพทยศาสตร์โรงพยาบาลรามาธิบดี มหาวิทยาลัยมหิดล

e-mail: Chatchalit.rat@mahidol.ac.th

โทร 081-7982529

ผู้ร่วมวิจัย 2 รศ. พญ. ดร.อติพร อิงค์สาธิต

Atiporn Ingsathit, M.D, PHD.

ตำแหน่ง รองศาสตราจารย์

คุณวุฒิ แพทยศาสตรบัณฑิต คณะแพทยศาสตร์โรงพยาบาลรามาธิบดี มหาวิทยาลัยมหิดล

วุฒิบัตรผู้ชำนาญในการประกอบวิชาชีพเวชกรรมสาขาอายุรศาสตร์ (แพทยสภา)

วุฒิบัตรผู้ชำนาญในการประกอบวิชาชีพเวชกรรมอนุสาขาอายุรศาสตร์โรคไต

ปริญญาเอกระบาดวิทยาคลินิก มหาวิทยาลัยมหิดล

สถานที่ทำงาน สาขาวิชาโรคไต ภาควิชาอายุรศาสตร์

คณะแพทยศาสตร์โรงพยาบาลรามาธิบดี มหาวิทยาลัยมหิดล

e-mail: Atiporn.ing@mahidol.ac.th

โทร 02-2012446

ผู้ร่วมวิจัย 3 น.ส. ฉัตรวรา อารีวุฒิ

Chatvara Areevut, MS, RD

ตำแหน่ง นักกำหนดอาหาร

คุณวุฒิ Master of Science

Registered Dietitian (US)

สถานที่ทำงาน สาขาวิชาต่อมไร้ท่อและเมแทบอลิซึม ภาควิชาอายุรศาสตร์

คณะแพทยศาสตร์โรงพยาบาลรามาธิบดี มหาวิทยาลัยมหิดล

e-mail: Chatvara244@yahoo.com

โทร: 090-2921505

ผู้ร่วมวิจัย 4 น.ส. สุนีย์ แซ่ตั้ง

Sunee Saetung, M.Sc.

ตำแหน่ง พยาบาล

คุณวุฒิ วิทยาศาสตรมหาบัณฑิต (อาหารและโภชนาการเพื่อการพัฒนา)

สถานที่ทำงาน สาขาวิชาต่อมไร้ท่อและเมแทบอลิซึม ภาควิชาอายุรศาสตร์

คณะแพทยศาสตร์โรงพยาบาลรามาธิบดี มหาวิทยาลัยมหิดล

e-mail: ssaetune@hotmail.com

โทร: 02-2011643

4. แพทย์และผู้ดูแลผู้เข้าร่วมวิจัย

4.1 พญ. อรณิชา สุนทรโลหะนะกูล

สถานที่ทำงาน สาขาวิชาต่อมไร้ท่อและเมแทบอลิซึม ภาควิชาอายุรศาสตร์
คณะแพทยศาสตร์โรงพยาบาลรามาธิบดี มหาวิทยาลัยมหิดล
e-mail: Onnicha.sun@gmail.com
โทร 087-7164646

ที่อยู่ (บ้าน) 11/26 ซ.ศูนย์วิจัย ถ.เพชรบุรีตัดใหม่ เขตห้วยขวาง
แขวง บางกะปิ กรุงเทพมหานคร 10310
โทร 02-314-2753

4.2 น.ส. ฉัตรวรา อารีวุฒิ

สถานที่ทำงาน สาขาวิชาต่อมไร้ท่อและเมแทบอลิซึม ภาควิชาอายุรศาสตร์
คณะแพทยศาสตร์โรงพยาบาลรามาธิบดี มหาวิทยาลัยมหิดล
e-mail: Chatvara244@yahoo.com
โทร: 090-2921505

ที่อยู่ (บ้าน) 246 ม.ฟ้าลากูน ต.ประชาธิปัตน์ อ.ธัญบุรี
จ.ปทุมธานี 12130
โทร 02-096-1159

5. หลักการและเหตุผล การทบทวนวรรณกรรม

โรคเบาหวานเป็นปัญหาสำคัญทางสาธารณสุขทั่วโลกเนื่องจากเป็นโรคเรื้อรัง และ ทำให้เกิดภาวะแทรกซ้อนหลายอวัยวะ คือ ตา (Diabetic retinopathy) ไต (Diabetic nephropathy) ระบบประสาท (Diabetic neuropathy) รวมถึงความเสี่ยงต่อภาวะโรคหัวใจหลอดเลือด (Cardiovascular disease) โรคหลอดเลือดแดงหัวใจโคโรนารี (Coronary artery disease) โรคหลอดเลือดสมอง (Cerebrovascular disease) และโรคหลอดเลือดส่วนปลายตีบ (Peripheral artery disease) ผู้ป่วยที่เป็นโรคเบาหวานมีอัตราการเกิดทุพพลภาพ และอัตราตายสูง

ความชุกของโรคเบาหวานเพิ่มมากขึ้นทั่วโลก โดยเฉพาะโรคเบาหวานชนิดที่ 2 การสำรวจสุขภาพประชาชนไทยโดยการตรวจร่างกาย ครั้งที่ 5 (Thai National Health Examination Survey, NHES V) โดย วิชัย เอกพลากร และคณะ[1] พบว่าใน พ.ศ.2557 ความชุกของผู้มีระดับน้ำตาลใน

เลือด (plasma glucose) ผิดปกติ และโรคเบาหวานในประชากรอายุตั้งแต่ 15 ปีขึ้นไปเท่ากับร้อยละ 8.96 เพิ่มขึ้นจากเดิมเมื่อ พ.ศ.2552 ซึ่งมีความชุกร้อยละ 6.9

ปัจจุบันมีข้อมูลที่ได้รับการพิสูจน์แล้วว่า หากรักษาโรคเบาหวานชนิดที่ 2 โดยคุมค่าน้ำตาลสะสม (Glycosylated hemoglobin; HbA1c) ให้อยู่ในค่าที่เหมาะสมสำหรับผู้ป่วยแต่ละราย โดยเฉลี่ยน้อยกว่าหรือเท่ากับร้อยละ 6.5-7 จะสามารถลดภาวะแทรกซ้อนทางตา ไต และอัตราการตายในระยะยาวได้ [2-6]

HbA1c เป็นผลจากระดับน้ำตาลในเลือดขณะอดอาหาร (Fasting Plasma Glucose; FPG) และระดับน้ำตาลในเลือดหลังรับประทานอาหาร (Post Prandial Plasma Glucose; PPPG) โดย plasma glucose แต่ละเวลามีผลต่อ HbA1c ไม่เท่ากัน การศึกษาโดย Monnier และคณะ[7] พบว่าผู้ป่วยที่มี HbA1c สูง FPG จะมีผลต่อ HbA1c มากกว่า PPPG ตัวอย่างเช่น ผู้ป่วยที่มี HbA1c มากกว่าร้อยละ 10.2 FPG จะมีผลต่อ HbA1c ถึงร้อยละ 70 แต่ PPPG จะมีผลต่อ HbA1c เพียงร้อยละ 30 ในทางกลับกันถ้าผู้ป่วยมี HbA1c สูงแต่ไม่มาก เช่น HbA1c น้อยกว่าร้อยละ 7.3 FPG จะมีผลต่อHbA1c เพียงร้อยละ 30 แต่ PPPG จะมีผลต่อ HbA1c ถึงร้อยละ 70 Riddle และคณะ[7] ได้ทำการศึกษาเพื่อสนับสนุนผลการศึกษานี้พบว่าผู้ป่วยที่ได้รับการรักษาด้วยอินซูลินแบบพื้นฐาน (basal insulin) และ FPG อยู่ในเกณฑ์ดี plasma glucose ที่มีผลต่อ HbA1c จะเป็น PPPG ร้อยละ 65-68

American Diabetes Association (ADA) 2017 [8] ได้ให้คำแนะนำในการรักษาเบาหวานชนิดที่ 2 โดยเริ่มจากให้ยาลดระดับน้ำตาล (oral hypoglycemic agent) จนครบ 3 ตัว และหากไม่สามารถรักษา HbA1c ให้อยู่ในเกณฑ์ที่เหมาะสมให้เริ่มฉีด basal insulin การปรับ basal insulin ให้พิจารณา FPG ว่าอยู่ในเกณฑ์หรือไม่ และให้เพิ่มขนาด basal insulin จน FPG อยู่ในเกณฑ์ที่เหมาะสม ถ้า FPG อยู่ในเกณฑ์ที่เหมาะสมแต่ HbA1c ยังไม่ถึงเป้าหมาย ให้พิจารณาแก้ไข PPPG โดยปฏิบัติดังนี้

1. ฉีดอินซูลินชนิดออกฤทธิ์สั้นก่อนอาหารมื้อหลัก 1 มื้อ เพิ่มจากการฉีด basal insulin (basal plus regimen)
2. เปลี่ยนมาฉีดอินซูลินชนิดผสม (pre-mixed insulin)
3. ฉีดยา GLP-1 agonist เพิ่มจากการฉีด basal insulin

เนื่องจากหลักปฏิบัติข้างต้นจำเป็นต้องฉีดยาผู้ป่วยอย่างน้อย 1 ครั้งไม่ว่าจะเป็น basal plus regimen หรือ ยา GLP-1 agonist เพิ่มความยุ่งยากให้ผู้ป่วยและเสี่ยงต่อการเกิดภาวะน้ำตาลต่ำในเลือด (hypoglycemia) นอกจากนี้ยากลุ่ม GLP-1 agonist ยังมีราคาแพง ทำให้ไม่สามารถปฏิบัติได้จริง

การศึกษาของ Diepietro และคณะ [9] พบว่าในบุคคลที่มีความเสี่ยงต่อการเกิดโรคเบาหวานชนิดที่ 2 ภายหลังรับประทานอาหารเสร็จ 30 นาที การเดินบนลู่วิ่งสายพาน (treadmill) ด้วยความเร็ว 3 METs (Metabolic Equivalent Tasks, MET value = VO_2 (mL (kg min)$^{-1}$)/3.5) (เทียบเท่าความเร็วเฉลี่ยประมาณ 4.83 กิโลเมตร/ชั่วโมง) เป็นเวลา 15 นาที สามารถลดระดับน้ำตาลเฉลี่ย 24 ชั่วโมง (24-hour mean plasma glucose) จาก 127 มก/ดล เป็น 115 มก/ดล หรือร้อยละ 10 เปรียบเทียบกับวันที่ไม่เดินอย่างมีนัยสำคัญทางสถิติ และการศึกษานี้ยังพบว่าการเดินด้วยความเร็วที่เท่ากันแต่เดินครั้งเดียวติดต่อกัน 45 นาที ในเวลาเช้าและเวลาบ่ายให้ผลที่ด้อยกว่าการเดินหลังอาหารครั้งละ 15 นาทีสำหรับระดับน้ำตาล 3 ชม.หลังอาหารมื้อเย็น (3-hour post dinner plasma glucose) คำนวณจากพื้นที่ใต้กราฟ (3-hour incremental area under the curve; iAUC) การศึกษานี้จึงสรุปได้ว่าในผู้ป่วยที่มีความเสี่ยงต่อการเป็นเบาหวานการเดินหลังอาหาร 3 มื้อเป็นเวลา 15 นาที ด้วยความเร็วเฉลี่ย 4.83 กิโลเมตร/ชั่วโมง ลด plasma glucose มากกว่าการเดินครั้งเดียวด้วยเวลาและความหนักที่เท่ากันแต่ไม่ใช่หลังมื้ออาหาร

การศึกษาของ Colberg และคณะ [10] เปรียบเทียบการเดิน 20 นาที ด้วยความเร็วเฉลี่ย 3.54 กิโลเมตร/ชั่วโมง ในลู่วิ่งสายพาน ก่อนรับประทานอาหารเย็น หลังรับประทานอาหารเย็น และไม่เดิน ในผู้ป่วยเบาหวานชนิดที่ 2 ผลการศึกษาพบว่าค่าเฉลี่ย 1-hour PPPG (Mean ± SEM) ของผู้ป่วยที่เดินหลังรับประทานอาหารมีค่าต่ำสุด ดังนี้ เดินหลังรับประทานอาหาร 131.4 ± 16.3 มก/ดล ไม่เดิน 172.8 ± 10.3 มก/ดล เดินก่อนรับประทานอาหาร 181.2 ± 16.4 มก/ดล นอกจากนี้หากเปรียบเทียบ 1-hour PPPG กับระดับน้ำตาลก่อนรับประทานอาหาร พบว่าการเดินหลังรับประทานอาหารสามารถลด plasma glucose ได้อย่างมีนัยสำคัญทางสถิติ (-3.4 ± 12.6 มก/ดล) เทียบกับการไม่เดิน (+23±13.4 มก/ดล) และการเดินก่อนรับประทานอาหาร (+31±13.5 มก/ดล) ตามลำดับ การศึกษานี้จึงสรุปได้ว่าการเดินหลังรับประทานอาหารด้วยความเร็วเฉลี่ย 3.54 กิโลเมตร/ชั่วโมง เป็นเวลา 20 นาที สามารถลด 1-hour PPPG ได้ประมาณ 40 มก/ดล ในขณะที่การเดินก่อนรับประทานอาหารเพิ่ม 1-hour PPPG

Dijk และคณะ [11] ศึกษาเปรียบเทียบการเดินเป็นระยะเวลา 15 นาที หลังรับประทานอาหารประมาณ 30 นาที ด้วยความเร็วเฉลี่ย 4.83 กิโลเมตร/ชั่วโมง กับการออกกำลังกาย 45 นาทีโดยการปั่นจักรยานตอนเช้าและการไม่เพิ่มกิจกรรมทางกาย โดยการคำนวณ iAUC ของระดับน้ำตาลหลังอาหาร 3.5 ชั่วโมง (3.5-hour PPPG) ผลการศึกษาพบว่า 3.5-hour PPPG ของมื้อเช้า กลางวันและเย็นในกลุ่มปั่นจักรยานมีค่าต่ำกว่ากลุ่มไม่เพิ่มกิจกรรมทางกายอย่างมีนัยสำคัญทางสถิติ (155±16 *vs.* 247±22 มิลลิโมล/ลิตร/3.5 ชม, 74±16 *vs.*113±17 มิลลิโมล/ลิตร/3.5 ชม, 55±16 *vs.* 89±22 มิลลิโมล/ลิตร/3.5 ชม ตามลำดับ) ส่วนการเดินหลังมื้ออาหารมีแนวโน้มลด 3.5-hour PPPG แต่ไม่มีนัยสำคัญทางสถิติหากเทียบกับกลุ่มไม่เพิ่มกิจกรรมทางกาย (208±23 มิลลิโมล/ลิตร/3.5 ชม,

101±19 มิลลิโมล/ลิตร/3.5 ชม, 56±15 มิลลิโมล/ลิตร/3.5 ชม ตามลำดับ) สรุปได้ว่าการเดินเป็นเวลา 15 นาทีหลังรับประทานอาหารมีแนวโน้มลด PPPG ได้

Reynolds และคณะ [12] ศึกษาเปรียบเทียบการเดินเป็นเวลา 10 นาที หลังรับประทานอาหาร 3 มื้อ กับการเดิน 30 นาทีต่อวัน ณ เวลาใดก็ได้ เป็นเวลา 2 สัปดาห์ ในผู้ป่วยเบาหวานชนิดที่ 2 ผลการศึกษาพบว่า PPPG 3 มื้อ 7 วันที่ติด Continuous Glucose Monitoring System (CGMS) คำนวณจาก iAUC ในกลุ่มที่เดินหลังมื้ออาหารมีค่าต่ำกว่ากลุ่มที่เดิน 30 นาทีต่อวันในเวลาใดก็ได้อย่างมีนัยสำคัญทางสถิติคิดเป็นร้อยละ 12 นอกจากนี้ผลดังกล่าวจะมีความแตกต่างมากที่สุดในอาหารมื้อเย็นซึ่งเป็นเวลาที่ผู้เข้าร่วมวิจัยรับประทานอาหารกลุ่มคาร์โบไฮเดรต และมีพฤติกรรมเนือยนิ่งมากที่สุด (sedentary lifestyle) การศึกษานี้สรุปได้ว่า การเดินเพียง 10 นาที หลังรับประทานอาหารสามารถลด PPPG ได้และลดได้มากกว่าการเดินโดยไม่กำหนดช่วงเวลา

เนื่องจากมีการศึกษาวิจัยยืนยันว่าการเดินหลังรับประทานอาหารเป็นเวลา 10-20 นาทีสามารถลด PPPG ได้มากกว่าการเดินก่อนรับประทานอาหาร และมีผลตั้งแต่ครั้งแรกที่เริ่มเดินแต่การศึกษาวิจัยส่วนใหญ่เป็นการการศึกษาระยะสั้น และอยู่ภายใต้การควบคุมของผู้วิจัย ยังไม่มีการศึกษาระยะยาวและยังไม่เคยมีการศึกษาการลดระดับน้ำตาลด้วยการเดินเทียบกับการรักษาเบาหวานด้วยวิธีอื่นที่ใช้ในปัจจุบัน เช่น oral hypoglycemic agent หรือ insulin

จึงเป็นที่มาของการศึกษาวิจัยว่าการเดินหลังรับประทานอาหารอย่างน้อย 1 มื้อ/วัน ทุกวันต่อสัปดาห์ ครั้งละ 15-20 นาที จะลด HbA1c และ PPPG ในผู้ป่วยเบาหวานชนิดที่ 2 ที่ได้ oral hypoglycemic agent และ basal insulin จน FPG อยู่ในเกณฑ์ที่เหมาะสม ไม่แพ้การรักษาด้วยวิธี basal plus regimen (basal insulin plus one dose premeal insulin) ซึ่งเป็นการรักษามาตรฐานของผู้ป่วยเบาหวานชนิดที่ 2 ที่ไม่สามารถควบคุมระดับน้ำตาลในเลือดได้ด้วย basal insulin เพียงอย่างเดียว [8]

6. วัตถุประสงค์ของโครงการวิจัย

6.1 **<u>วัตถุประสงค์หลัก (primary outcome)</u>** เพื่อศึกษาผลของการเดินหลังรับประทานอาหารเทียบกับ basal plus regimen ต่อระดับ HbA1c

<u>สมมติฐาน</u> : การเดินหลังรับประทานอาหารอย่างน้อย 1 มื้อ/วัน เป็นเวลา 15-20 นาทีทุกวันต่อสัปดาห์ ติดต่อกัน 6 สัปดาห์ สามารถลดระดับ HbA1c ไม่ต่างกับ basal plus regimen

6.2 **<u>วัตถุประสงค์รอง (secondary outcome)</u>** เพื่อศึกษาผลของการเดินหลังรับประทานอาหารเทียบกับ basal plus regimen ต่อค่าตัวแปรดังต่อไปนี้

1. Serum fructosamine

2. ค่าเฉลี่ยของระดับน้ำตาลหลังมื้ออาหาร 2 ชั่วโมง (2-hour mean PPPG) วัดจาก ผลการเจาะน้ำตาลปลายนิ้วด้วยตัวเองระหว่างอยู่ที่บ้าน (Self-Monitoring Blood Glucose; SMBG) ด้วยเครื่องเจาะตรวจระดับน้ำตาล (glucometer)
3. ค่าเฉลี่ยของระดับน้ำตาล 24 ชั่วโมง (24-hour mean plasma glucose) วัดจาก ผล SMBG ด้วย glucometer
4. PPPG และ Triglyceride (TG) ชั่วโมงที่ 1,2,3 และ 4 หลังรับประทาน standardized meal
5. อัตราการเกิดภาวะน้ำตาลต่ำในเลือด (hypoglycemia)
6. น้ำหนัก เส้นรอบเอว และค่าดัชนีมวลกาย (Body mass index, BMI)

<u>นิยามของภาวะน้ำตาลต่ำในเลือด</u>[9]

แบ่งเป็น 3 ระดับดังนี้

- Glucose alert value (level 1): plasma glucose $\leq$ 70 mg/dL
- Clinically significant hypoglycemia (level 2): plasma glucose < 54 mg/dL
- Severe hypoglycemia (level 3): มีอาการทางระบบประสาทอันเนื่องจาก hypoglycemia และจำเป็นต้องได้รับความช่วยเหลือจากบุคคลอื่น

7. วิธีวิจัยและแบบแผนการวิจัย ตารางการทำวิจัย

7.1 Study design: Randomized controlled cross-over study

7.2 สถานที่การทำวิจัย: คณะแพทยศาสตร์ โรงพยาบาลรามาธิบดี

<u>การทำวิจัยในแต่ละขั้นตอน</u>

<u>Assessed for eligibility and Run-in period (2 **สัปดาห์**)</u>

1. คณะผู้วิจัยทำการรวบรวมข้อมูลจากเวชระเบียนและเวชระเบียนอิเลคทรอนิกส์ สืบค้นประวัติการรักษาพยาบาล และ รวบรวมข้อมูลทางคลินิก ได้แก่
    1) ข้อมูลทั่วไป เช่น เพศ อายุ อาชีพ
    2) ข้อมูลเกี่ยวกับโรคเบาหวานชนิดที่ 2 ดังนี้
        i. ช่วงเวลา (duration) และ อายุ (age at diagnosis) ที่ได้รับการวินิจฉัย
        ii. ภาวะแทรกซ้อน (complication)
        iii. ยาที่ใช้ในการรักษาภายใน 6 เดือน และขณะปัจจุบัน
        iv. HbA1c และ FBS ภายใน 6 เดือน

3) โรคร่วม (co-morbidity) เช่น โรคความดันเลือดสูง โรคไขมันในเลือดสูง
4) การดื่มสุรา หรือสูบบุหรี่
5) น้ำหนัก ส่วนสูงปัจจุบัน ค่าดัชนีมวลกาย และเส้นรอบเอว (waist circumference) ก่อนทำการศึกษา

2. ช่วง Run-in period ระยะเวลา 2 สัปดาห์ ปฏิบัติดังนี้
1) แจก glucose meter ให้ผู้เข้าร่วมวิจัย และศึกษาวิธีการใช้จากพยาบาลผู้เชี่ยวชาญ
2) แพทย์ให้ผู้เข้าร่วมวิจัยทบทวนการฉีด basal insulin วิธีการเก็บรักษา และการแก้ไข hypoglycemia ด้วยตัวเอง
3) ผู้เข้าร่วมวิจัยเรียนโภชนาการจากนักกำหนดอาหารผู้เชี่ยวชาญ (registered dietitian) เรียนวิธีจดบันทึกอาหารและอาหารว่าง ลงในรายการบันทึกอาหาร (food diary)
4) ผู้เข้าร่วมวิจัยได้รับเครื่องนับก้าวเดิน (accelerometer) และเรียนรู้วิธีการใช้อุปกรณ์ดังกล่าว
5) ผู้เข้าร่วมวิจัยทดลองเจาะ SMBG วันละ 6 ครั้ง ก่อนอาหาร 3 มื้อ คือ อาหารเช้า กลางวัน เย็น และหลังอาหารที่เวลา 2 ชม 1 วัน/สัปดาห์ เป็นเวลา 2 สัปดาห์
6) ผู้เข้าร่วมวิจัยบันทึกรายการอาหารที่รับประทานในรายการบันทึกอาหาร วันที่เจาะ SMBG และให้ registered dietitian พิจารณาเมื่อครบสัปดาห์แรก
7) ในสัปดาห์แรกผู้เข้าร่วมวิจัยต้องติดเครื่อง accelerometer ทุกวัน เพื่อประเมินจำนวนก้าวที่เดินในแต่ละวัน ขณะดำเนินชีวิตประจำวันตามปกติ
8) ในสัปดาห์ที่สอง ผู้เข้าร่วมวิจัยต้องทดลองเดินหลังรับประทานอาหารเสร็จประมาณ 15-30 นาที โดยเดินให้เร็วที่สุดเท่าที่จะเดินได้ เป็นเวลา 15-20 นาที อย่างน้อย 1 มื้ออาหาร/วัน 7 วันต่อสัปดาห์ โดยติดเครื่อง accelerometer เพื่อยืนยันว่าสามารถปฏิบัติภาวะดังกล่าวจริง

หลังจบ Run-in period ผู้เข้าร่วมวิจัยจะถูกแบ่งเป็น 2 กลุ่มโดยการสุ่มแบบ 1:1 และได้รับการเจาะเลือด ตรวจปัสสาวะ ดังรายการต่อไปนี้ (การเจาะเลือดนับเป็นการเจาะเลือดของสัปดาห์ที่ 0 ของแต่ละกลุ่ม)

1) HbA1c
2) FPG
3) Serum fructosamine

4) Fasting lipid profile
5) วัด PPPG และ TG ชั่วโมงที่ 1,2,3 และ 4 หลังรับประทาน standardized meal

<u>กลุ่มที่ 1: Post meal walking group (ระยะเวลา 6 สัปดาห์)</u>

1. ผู้เข้าร่วมวิจัยรับประทาน oral hypoglycemic agent และฉีด basal insulin ตามปกติ
2. ผู้เข้าร่วมวิจัยต้องเดินหลังรับประทานอาหารเสร็จ ประมาณ 15-30 นาที โดยเดินให้เร็วที่สุดเท่าที่จะเดินได้ เป็นเวลา 15-20 นาที อย่างน้อย 1 มื้ออาหาร / วัน เป็นเวลา 7 วันต่อสัปดาห์ โดยเลือกเดินหลังมื้ออาหารที่ผู้เข้าร่วมวิจัยถือว่าเป็นมื้อหลัก คือรับประทานอาหารมากกว่ามื้ออื่น และผู้เข้าร่วมวิจัยต้องติดเครื่อง accelerometer ยืนยันว่าปฏิบัติภาวะดังกล่าวจริง
3. ผู้เข้าร่วมวิจัยต้องทำ SMBG 1 วันต่อสัปดาห์ โดยเจาะก่อนอาหารและหลังอาหาร 2 ชม 3 มื้อ คือ มื้อเช้า มื้อกลางวัน มื้อเย็น รวมเป็น 6 ครั้ง
4. ในขณะดำเนินชีวิตประจำวัน ผู้เข้าร่วมวิจัยต้องติดเครื่อง accelerometer ทุกวัน เพื่อประเมินจำนวนก้าวที่เดิน
5. ผู้เข้าร่วมวิจัยต้องไม่ออกกำลังกายเพิ่มจากปกติระหว่างอยู่ในงานวิจัยกลุ่ม post meal walking group
6. ผู้เข้าร่วมวิจัยต้องจดบันทึกอาหารที่รับประทานในแต่ละมื้อรวมถึงอาหารว่าง ลงในรายการบันทึกอาหาร ในวันที่ทำ SMBG
7. ผู้เข้าร่วมวิจัยพบแพทย์ผู้ดูแลที่สัปดาห์ที่ 3 เพื่อทบทวนการใช้ glucose meter และ accelerometer รวมทั้งเจาะเลือดดู serum fructosamine และ FPG
8. ผู้เข้าร่วมวิจัยพบแพทย์ผู้ดูแลที่สัปดาห์ที่ 6 เพื่อ
    1) เจาะเลือดดู serum fructosamine FPG HbA1c และ fasting lipid profile
    2) เจาะเลือดดู PPPG และ TG ชั่วโมงที่ 1,2,3 และ 4 หลังรับประทาน standardized meal

<u>กลุ่มที่ 2: Basal plus group (ระยะเวลา 6 สัปดาห์)</u>

1. ผู้เข้าร่วมวิจัยรับประทาน oral hypoglycemic agent และฉีด basal insulin ตามปกติ
2. ผู้เข้าร่วมวิจัยต้องฉีดอินซูลินชนิดออกฤทธิ์เร็ว (rapid acting insulin) ก่อนอาหารมื้อหลัก 15 นาที โดยอาหารมื้อหลัก คือ มื้อที่ผู้เข้าร่วมวิจัยรับประทานอาหารมากกว่ามื้ออื่น สำหรับขนาดของ rapid acting insulin เริ่มที่ 4 ยูนิต/มื้อ หรือ 0.1 ยูนิต/น้ำหนักตัว 1 กิโลกรัม โดยแพทย์ผู้ดูแลผู้เข้าร่วมงานวิจัยจะผู้เป็นพิจารณาขนาดของอินซูลิน

3. ผู้เข้าร่วมวิจัยต้องทำ SMBG ก่อนและหลังรับประทานอาหารมื้อหลักที่ 2 ชั่วโมง ติดกัน 3 วัน เพื่อปรับขนาด rapid acting insulin ให้ 2-hour PPPG ≤180 มก/ดล (การปรับอินซูลินนี้ แพทย์จะปรับผ่านทางอุปกรณ์อิเล็กทรอนิกส์ เช่น โทรศัพท์มือถือ, line application จนกว่า 2-hour PPPG ≤180 มก/ดล ติดต่อกัน 2 ครั้ง) เมื่อ 2-hour PPPG ≤180 มก/ดล ผู้เข้าร่วมวิจัยต้องทำ SMBG 1 วัน ต่อสัปดาห์ โดยเจาะก่อนอาหารและหลังอาหาร 2 ชม 3 มื้อ คือ มื้อเช้า กลางวัน เย็น
4. ในขณะดำเนินชีวิตประจำวัน ผู้เข้าร่วมวิจัยต้องติดเครื่อง accelerometer ทุกวัน เพื่อประเมินจำนวนก้าวที่เดิน
5. ผู้เข้าร่วมวิจัยต้องไม่เดินหลังรับประทานอาหาร และไม่ออกกำลังกายเพิ่มจากปกติระหว่างอยู่ในงานวิจัยกลุ่ม basal plus group
6. ผู้เข้าร่วมวิจัยต้องจดบันทึกอาหารที่รับประทานในแต่ละมื้อ รวมถึงอาหารว่างลงรายการบันทึกอาหารในวันที่ทำ SMBG
7. ผู้เข้าร่วมวิจัยพบแพทย์ผู้ดูแลที่สัปดาห์ที่ 3 เพื่อทบทวนการใช้ glucose meter และ accelerometer รวมทั้งเจาะเลือดดู serum fructosamine และ FPG
8. ผู้เข้าร่วมวิจัยพบแพทย์ผู้ดูแลที่สัปดาห์ที่ 6 เพื่อ
    1) เจาะเลือดดู serum fructosamine FPG HbA1c และ fasting lipid profile
    2) เจาะเลือดดู PPPG และ TG ชั่วโมงที่ 1,2,3 และ 4 หลังรับประทาน standardized meal
    3) รับการสัมภาษณ์โดยแบบสอบถามเพื่อประเมินกิจกรรมทางกาย International physical activity questionnaire (IPAQ) [14]

<u>Wash out period (**ระยะเวลา 2 สัปดาห์**)</u>

หลังสิ้นสุดการเข้าร่วมวิจัยแต่ละกลุ่ม ผู้เข้าร่วมวิจัยจะเข้าสู่ wash out period 2 สัปดาห์ ในช่วงนี้ผู้เข้าร่วมวิจัยต้องปฏิบัติตนเสมือนก่อนเข้าร่วมงานวิจัย คือ รับประทาน oral hypoglycemic agent ฉีด basal insulin ตามปกติ เดินตามปกติ ไม่เดินหลังรับประทานอาหาร และเมื่อครบระยะเวลาดังกล่าวผู้เข้าร่วมวิจัยจะต้องเจาะเลือดนับเป็นการเจาะเลือดของสัปดาห์ที่ 0 ของกลุ่มวิจัยกลุ่มถัดไป โดยเจาะเลือดดังต่อไปนี้

1) HbA1c
2) FPG
3) Serum fructosamine
4) Fasting lipid profile

5) วัด PPPG และ TG ชั่วโมงที่ 1,2,3 และ 4 หลังรับประทาน standardized meal

<u>เครื่องมือ และขั้นตอนการวิจัย</u>

1. **การวัด PPPG และ TG หลังรับประทาน** standardized meal
    - ทำตอนเช้าหลังอดอาหารและน้ำข้ามคืนเป็นเวลา 12 ชั่วโมง
    - นาทีที่ 0 เจาะเลือดส่งตรวจ plasma glucose และ fasting lipid profile
    - จากนั้นให้รับประทาน Isocal (liquid meal) 250 ซีซี ซึ่งมีพลังงาน 256 แคลอรี ประกอบด้วยไขมัน ร้อยละ 37 คาร์โบไฮเดรตร้อยละ 50 และโปรตีนร้อยละ 13 ที่ชั่วโมงที่ 1,2,3 และ 4 เจาะเลือดส่งตรวจ plasma glucose และ TG
2. Accelerometer
    - Tri-axial accelerometer บริษัท Fitbit รุ่น Fitbit Zip
    - เป็นเครื่อง accelerometer ที่กันน้ำได้ สามารถเก็บข้อมูลได้มากสุดที่ 7 วัน และส่งผ่านข้อมูลผ่าน wireless เข้าโทรศัพท์มือถือได้
    - สามารถติดที่บริเวณเข็มขัด กางเกง หรือ ชุดชั้นใน ของผู้เข้าร่วมงานวิจัย

Fitbit Zip ได้รับการศึกษาถึงความเที่ยงตรง (validity) ในบุคคลหลายกลุ่ม และหลายประเทศ [13-19]

3. **เครื่องเจาะน้ำตาล บริษัท** Abbot **รุ่น** Freestyle optium H
4. **การเจาะเลือด**
    - HbA1c ใช้วิธี Turbidimetric inhibition immunoassay (TINIA) บริษัท Roche
    - Plasma glucose ใช้วิธี Hexokinase/Glucose-6-Phosphate Dehydrogenase (HK/G6P-DH) บริษัท Abbot
    - Serum fructosamine ใช้วิธี chemiluminescence บริษัท Roche
    - Fasting lipid profile ใช้วิธี accelerator selective dependent method บริษัท Abbot

8. Protocol Flow Chart

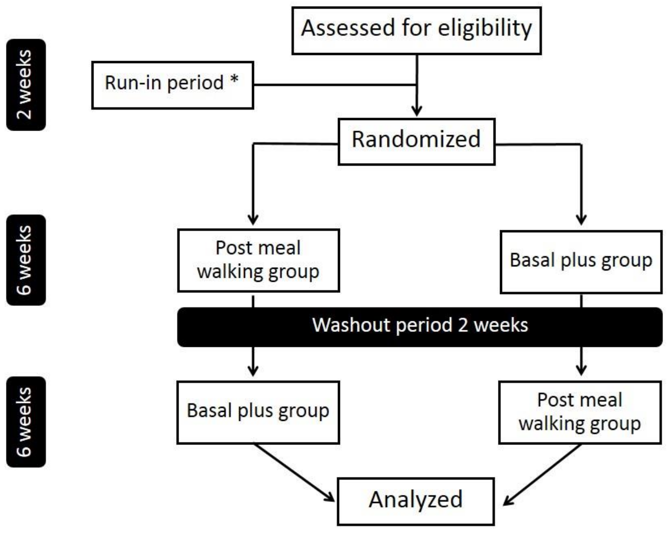

แผนภูมิที่ 1 แสดงแนวทางการทำวิจัย

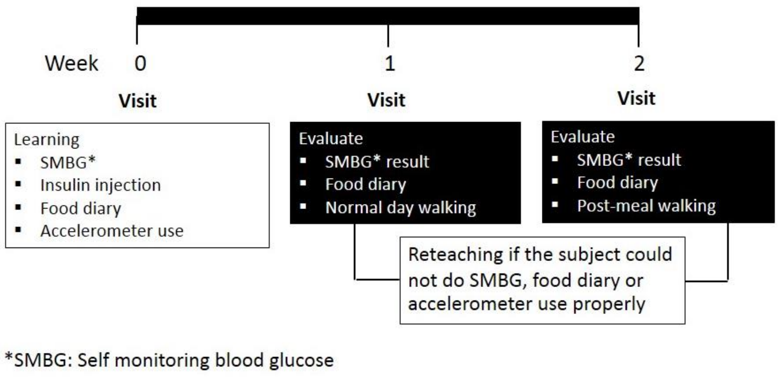

แผนภูมิที่ 2 แสดงแนวทางการดูแลผู้ป่วยในช่วง Run-in period

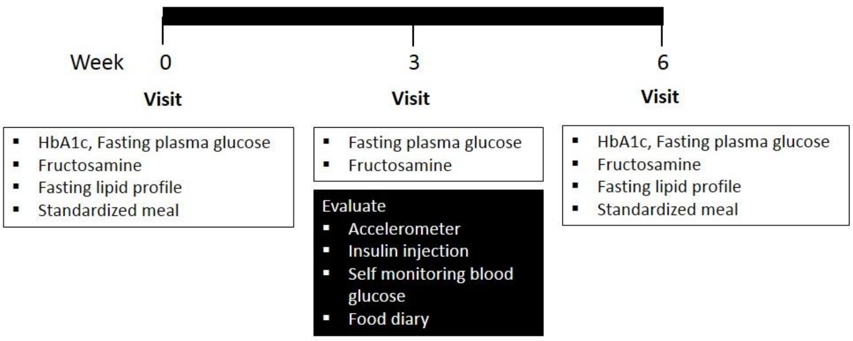

Follow up diagram in both groups

แผนภูมิที่ 3 แสดงระยะเวลาติดตามร่วมกับการตรวจทางห้องปฏิบัติการขณะเข้าร่วมวิจัย

9.ระบุจำนวนผู้เข้าร่วมการวิจัย (Subject) ที่จะศึกษา พร้อมทั้งเกณฑ์คัดเข้าและเกณฑ์คัดออก

9.1 วิธีการคำนวณกลุ่มตัวอย่าง

ใช้สูตรสำหรับ hypothesis testing[20] และเป็น two dependent means (for pair-matched study) โดยตั้งวัตถุประสงค์หลัก (primary outcome) เป็น HbA1c

$$n = \frac{(z_{1-\frac{\alpha}{2}} + z_{1-\beta})^2 \sigma^2}{\Delta^2}$$

โดยตัวแปรมีค่าดังนี้

1. σ คือ standard deviation ของข้อมูล อ้างอิงจากการศึกษาของ Lankisch และคณะ [21] สำหรับกลุ่ม basal plus group ได้ค่า standard deviation ของ HbA1c ที่ 0.65
2. α คือ โอกาสที่จะเกิด type I error ให้ค่าที่ 0.05
3. β คือ โอกาสที่จะเกิด type II error ให้ค่าที่ 0.80
4. Δ (Delta) คือ ความแตกต่างของ primary outcome ระหว่างสองกลุ่ม ให้ค่าเป็น 0.5 เนื่องจากถือว่า HbA1c ที่ต่างกัน 0.5 มีความสำคัญทางคลินิก

เมื่อคำนวณจากสูตรข้างต้นจะคำนวณกลุ่มตัวอย่างได้ 14 ราย และคาดว่าจะมีผู้วิจัยออกจากการศึกษาประมาณ 20% จึงตั้งเป้าหมาย จำนวนกลุ่มตัวอย่างตั้งต้นไว้ 16 ราย

<u>เกณฑ์การคัดเลือกผู้เข้าร่วมวิจัย</u> <u>(Inclusion criteria)</u>

1. ผู้ป่วยเบาหวานชนิดที่ 2 อายุ 35-70 ปี ที่ได้รับการรักษาด้วย oral hypoglycemic agent อย่างน้อย 2 ชนิด คู่กับการฉีด basal insulin (NPH, Glargine, Determir, Degludec)

2. FPG < 150 มก/ดล และ HbA1c อยู่ระหว่างร้อยละ 7-9
3. ค่าดัชนีมวลกาย อยู่ระหว่าง 18.5-30 กิโลกรัม/ตารางเมตร^2
4. ยินดีเข้าร่วมโครงการโดยการลงนาม

<u>เกณฑ์การคัดออกผู้เข้าร่วมวิจัย (Exclusion criteria)</u>

1. มีความดันเลือดสูงที่รุนแรง (systolic blood pressure > 160 mmHg or diastolic blood pressure > 100 mmHg)
2. เป็นโรคกล้ามเนื้อหัวใจขาดเลือดเฉียบพลัน หรือโรคหลอดเลือดในสมองภายใน 3 เดือน
3. เป็นโรคปอด หรือ โรคหัวใจวายเรื้อรัง (functional class II) ไม่สามารถเดินได้
4. เป็นโรคทางกระดูก ข้อหรือ มีเท้าผิดรูปเป็นอุปสรรคต่อการเดิน
5. มีปัญหาทางสายตา มีอุปสรรคต่อการมองเห็น
6. มีปัญหาเกี่ยวกับปลายประสาทส่วนปลาย คือ มีเท้าชา และตรวจร่างกายพบ decrease pinprick sensation
7. เคยมีแผลที่เท้า (diabetic foot ulcer) รุนแรง ต้องนอนโรงพยาบาล หรือเคยได้รับการตัดอวัยวะส่วนปลายของขา (amputation)
8. ได้รับการรักษาด้วยสารสเตียรอยด์
9. มีประวัติใช้สารเสพติดหรือมีความผิดปกติทางระบบประสาท
10. ดื่มเครื่องดื่มแอลกอฮอล์ ปริมาณมากกว่า 7 drinks ต่อสัปดาห์ (1 drink เท่ากับ เบียร์ 1 กระป๋อง, ไวน์ 1 แก้ว, เหล้า 40 ดีกรี 1 เป๊ก)
11. ทำงานกะกลางคืน หรือกะหมุนเวียน
12. กำลังตั้งครรภ์
13. ได้ยาฉีดอินซูลินชนิดอื่นๆ เช่น pre-mixed insulin
14. ได้ยา GLP-1 agonist
15. ได้ยาลดระดับน้ำตาลในเลือดกลุ่ม alpha-glucosidase inhibitor
16. ผู้เข้าร่วมวิจัยปฏิเสธหรือขอถอนตัว

ผู้เข้าร่วมวิจัยจะถูกเพิกถอนจากงานวิจัย เมื่อมีภาวะดังต่อไปนี้

1. ผู้เข้าร่วมงานวิจัยที่มี diabetic ketoacidosis หรือ hyperglycemic coma
2. ผู้เข้าร่วมงานวิจัยที่มี FPG > 250 mg/dL ติดต่อกัน 2 ครั้ง ระหว่างอยู่ในงานวิจัย (มีความจำเป็นต้องเปลี่ยน insulin regimen)

3. ผู้เข้าร่วมงานวิจัยที่มี HbA1c > 9 % ระหว่างอยู่ในงานวิจัย (มีความจำเป็นต้องเปลี่ยน insulin regimen)

10.การวิเคราะห์ทางสถิติ

ทำการเก็บข้อมูลโดยใช้โปรแกรม Epidata version 3.1

วิเคราะห์ข้อมูลด้วยโปรแกรม Stata 15

เอกสารอ้างอิง

1. ศ.นพ. วิชัย เอกพลากร และคณะ 2559;Pageshttp://kb.hsri.or.th/dspace/handle/11228/4604.
2. Intensive blood-glucose control with sulphonylureas or insulin compared with conventional treatment and risk of complications in patients with type 2 diabetes (UKPDS 33). UK Prospective Diabetes Study (UKPDS) Group. Lancet. 1998;352(9131):837-53.
3. Gerstein HC, Miller ME, Byington RP, Goff DC, Jr., Bigger JT, Buse JB, et al. Effects of intensive glucose lowering in type 2 diabetes. N Engl J Med. 2008;358(24):2545-59.
4. Holman RR, Paul SK, Bethel MA, Matthews DR, Neil HA. 10-year follow-up of intensive glucose control in type 2 diabetes. N Engl J Med. 2008;359(15):1577-89.
5. Patel A, MacMahon S, Chalmers J, Neal B, Billot L, Woodward M, et al. Intensive blood glucose control and vascular outcomes in patients with type 2 diabetes. N Engl J Med. 2008;358(24):2560-72.
6. Duckworth W, Abraira C, Moritz T, Reda D, Emanuele N, Reaven PD, et al. Glucose control and vascular complications in veterans with type 2 diabetes. N Engl J Med. 2009;360(2):129-39.
7. Riddle M, Umpierrez G, DiGenio A, Zhou R, Rosenstock J. Contributions of basal and postprandial hyperglycemia over a wide range of A1C levels before and after treatment intensification in type 2 diabetes. Diabetes Care. 2011;34(12):2508-14.
8. Standards of Medical Care in Diabetes-2017. Diabetes Care. 2017;40(Suppl 1):S1-S135.
9. DiPietro L, Gribok A, Stevens MS, Hamm LF, Rumpler W. Three 15-min bouts of moderate postmeal walking significantly improves 24-h glycemic control in older people at risk for impaired glucose tolerance. Diabetes Care. 2013;36(10):3262-8.
10. Colberg SR, Zarrabi L, Bennington L, Nakave A, Thomas Somma C, Swain DP, et al. Postprandial walking is better for lowering the glycemic effect of dinner than pre-dinner exercise in type 2 diabetic individuals. J Am Med Dir Assoc. 2009;10(6):394-7.
11. van Dijk JW, Venema M, van Mechelen W, Stehouwer CD, Hartgens F, van Loon LJ. Effect of moderate-intensity exercise versus activities of daily living on 24-hour blood glucose homeostasis in male patients with type 2 diabetes. Diabetes Care. 2013;36(11):3448-53.
12. Reynolds AN, Mann JI, Williams S, Venn BJ. Advice to walk after meals is more effective for lowering postprandial glycaemia in type 2 diabetes mellitus

than advice that does not specify timing: a randomised crossover study. Diabetologia. 2016;59(12):2572-8.
13. An HS, Jones GC, Kang SK, Welk GJ, Lee JM. How valid are wearable physical activity trackers for measuring steps? Eur J Sport Sci. 2016:1-9.
14. Case MA, Burwick HA, Volpp KG, Patel MS. Accuracy of smartphone applications and wearable devices for tracking physical activity data. Jama. 2015;313(6):625-6.
15. Ferguson T, Rowlands AV, Olds T, Maher C. The validity of consumer-level, activity monitors in healthy adults worn in free-living conditions: a cross-sectional study. Int J Behav Nutr Phys Act. 2015;12:42.
16. Lee JM, Kim Y, Welk GJ. Validity of consumer-based physical activity monitors. Med Sci Sports Exerc. 2014;46(9):1840-8.
17. Schneider M, Chau L. Validation of the Fitbit Zip for monitoring physical activity among free-living adolescents. BMC Res Notes. 2016;9(1):448.
18. Tully MA, McBride C, Heron L, Hunter RF. The validation of Fibit Zip physical activity monitor as a measure of free-living physical activity. BMC Res Notes. 2014;7:952.
19. Kooiman TJ, Dontje ML, Sprenger SR, Krijnen WP, van der Schans CP, de Groot M. Reliability and validity of ten consumer activity trackers. BMC Sports Sci Med Rehabil. 2015;7:24.
20. Chow S-C, Shao,J., Wang, H. Sample Size Calculations in Clinical Research Chapman & Hall/CRC; 2003.
21. Lankisch MR, Ferlinz KC, Leahy JL, Scherbaum WA. Introducing a simplified approach to insulin therapy in type 2 diabetes: a comparison of two single-dose regimens of insulin glulisine plus insulin glargine and oral antidiabetic drugs. Diabetes Obes Metab. 2008;10(12):1178-85.
