# Supplementary material for: Glycemic effect of post-meal walking compared to one prandial insulin injection in type 2 diabetic patients treated with basal insulin: A randomized controlled cross-over study
Source: PLoS One. 2020 Apr 1;15(4):e0230554. doi: 10.1371/journal.pone.0230554 (PMC7112182; doi:10.1371/journal.pone.0230554)
Supplement: Attachment — Submitted filename: Responses to reviewers letter.docx [file pone.0230554.s010.docx]

Manuscript PONE-D-19-20612

**Responses to reviewer #1**

1. The study was only over a very short period. HbA1c will not have had time to change as significantly as it could have over say 12 weeks.

**Answer:** It is well known that HbA1c is an estimate of mean plasma glucose levels in the previous 3-4 months, however 50% of the result is from mean plasma glucose level 0-30 days or 1 month prior to blood sampling (Can J Diabetes 2013; 37: S35-S39). Therefore we believe 6-week of intervention should have significant change (if any) to HbA1c results. We’re afraid that if the intervention of interest (post-meal walking) is continued for 12 weeks to capture all changes of HbA1c, the participants might not cooperate and we might have lost some of the participating subjects during the trial.

2. This was a group of patients were on a variety of OHDs – I realise that they were controls against themselves but I am surprised that some patients remained on sulphonylureas. I assume that the basal insulin (type and dosage) remained the same for both legs.

**Answer:** It is a common practice in Thailand that sulphonylureas is not withdrawn while being on basal insulin. As shown in the S3 table, basal insulin type was not changed. Basal insulin dosage was stable in all except two subjects in each arm, the dosage was minimally reduced due to mild hypoglycemia.

3. Only 14 out of 19 completed the study. Within these groups some in the walking post-meal group did not walk and is there any guarantee that in the group that had the prandial insulin that they did not walk post meal?

**Answer:** Since all participants carried accelerometers during the trial, we could monitor whether they walked after prandial insulin injection or not. It was shown that none of the participants in the basal plus arm violated the protocol. We have added this statement in the Results.

4. 700 steps is not very far and I suspect rather than aiming for “Walk as fast as possible” setting a target of possibly 1000 or 1500 steps would have been better.

**Answer:** We absolutely agree with you. The greater the number of post-meal steps, the lower of post-meal glucose levels is anticipated but in real life, walking for 1,000-1,500 steps post-meal might not be practical for some patients particularly with elderly patients. We try to make our protocol feasible and practical. However, we re-analyzed individual data of each participant and found that those who complied with the walking protocol, the averaged post-meal walking steps was in fact more than 1,000 steps as shown in S3 Table.

5. I am not sure why the time that patients were asked to walk was not after the same meal that they were going to take their prandial insulin – surely that would have been a better comparison?

**Answer:** We absolutely agree with this comment but in real life, the free time that patients have to spend on walking does not always get along with the main meal. As having been said, we try to make it practical.

6. I may have missed this - did any of the patients develop DKA or HHS?

**Answer:** None of the participants developed hyperglycemic emergencies during the trial

7. Only fructosamine and HbA1c were measured. Lipids, BP, weight etc would have been useful. Particularly a Q of L questionnaire would have been interesting to see if patients preferred the walking or the prandial insulin.

**Answer:** Unfortunately we did not collect those data

8. It would have better if the groups could have been controlled for calorific intake. Eating more at lunch in the walking group will have diluted the results.

**Answer:** We agree but unlike in clinical research environment, controlling for caloric intake in free living condition is difficult and make the study protocol more complicated

**Responses to reviewer#2**

Major concerns:
Although the authors address an important and relevant question, the study design is flawed by the duration of the study periods and the primary endpoint (A1c) chosen. The authors acknowledge that their design with 6 weeks treatment periods and a 2 weeks washout-period carries a significant risk of carry-over effects. The discussed fact, that the A1c did not change during the washout period it is not helpful at all since even significant changes in glycemic excursions are unlikely to change A1c concentrations within this short time period. Since the authors seem to be aware of this, either a different primary endpoint or a different study design should have been chosen.

**Answer:** As similar to our responses to reviewer#1, we believe 6-week of intervention should have significant change to HbA1c results since half of A1c change would occur in the first 30 days prior to blood sampling although it cannot capture all changes (Can J Diabetes 2013; 37: S35-S39). Fructosamine level which can reflect the shorter term changes of mean plasma glucose levels may be a better choice in this regard but since its measurement method is not well standardized and it is not a standard measure of glucose control in clinical practice, therefore we decide to use fructosamine as a secondary endpoint of the study.

Minor concern

1. Statistical analysis: the details of the the mixed model (i.e. parameters chosen for fixed, random effects, period, etc.) should be indicated in order to make the analysis plan reproducible.

**Answer:** The details of the mixed model statistical analysis were added in the manuscript as suggested

2. The patients were instructed to use prandial insulin before the main meal (basal plus period) or to briskly walk after at least one meal (post-meal walking period). To judge the comparative effects of the 2 interventions comparable time periods should be analyzed. I.e., if prandial insulin was given before dinner, this period should also be looked at in the post meal walking group and data should be presented accordingly.

**Answer:** As you can see in the S3 Table, the meals at which the patients walked or injected prandial insulin were not always the same, therefore it is impossible to analyze such data. We designed our protocol to be practical in real life, so we did not fix the meal at which post-meal walking was performed.

3. Detailed data regarding the insulin therapy should be indicated (i.e. dose, time-course of dose escalation) should be indicated in order to judge whether appropriate dose adjustments were made.

**Answer:** Insulin dose and changes of plasma glucose levels post-meal by walking or prandial insulin were demonstrated in S3 Table

4. The caloric intake in the basal plus group was significantly lower. Can the authors offer an explanation for this finding?

**Answer:** This finding may occur by chance since we did not control the caloric intake of the participants during the trial.

5. The individual participant data used for the final analysis should be made available.

**Answer:** The individual participant data was shown in S3 Table

**Other inquiries**

Please include a copy of Table 2 which you refer to in your text on page 11.

**Answer:** It is a mistake, Table 2 is actually a S2 Table. This has been corrected in the revised manuscript.

Please note that we have corrected some mistake in the abstract (line 33 page 2).

The study protocol has been deposited to protocol io and its DOI has been added in the manuscript as recommended.
