# Supplementary material for: Glycemic effect of post-meal walking compared to one prandial insulin injection in type 2 diabetic patients treated with basal insulin: A randomized controlled cross-over study
Source: PLoS One. 2020 Apr 1;15(4):e0230554. doi: 10.1371/journal.pone.0230554 (PMC7112182; doi:10.1371/journal.pone.0230554)
Supplement: Attachment — Submitted filename: Responses to reviewers letter_2nd revision.docx [file pone.0230554.s011.docx]

Manuscript PONE-D-19-20612_ 2^nd^ revision

**To the Editor**

The manuscript has been revised according to the reviewers’ suggestion, please be noted that the Fig 2 has been removed and replaced with Fig 3.

**Responses to reviewer #1.** The concept of exercise in place of insulin is an interesting and useful one. This should be regarded as a pilot and encourage others to undertake further work. There are faults with the design in this study but cannot be changed now.
The authors answer all the points made by the reviewers.

**Answer:** No specific query raised by the reviewer.

**Responses to reviewer#2.** Although the authors acknowledge the major concerns of the reviewers in their response letter no appropriate changes have been made to the manuscript. The authors should clearly state in their conclusion that the study results may be influenced by carry over effects due to the short intervention and wash-out periods chosen.

**Answer:** We have addressed the concerning issue raised by reviewer #2 in the Discussion part.

**Responses to reviewer#3.**

1. Abstract: Please clarify the following statement since both confidence intervals contain zero. It seems reasonable that neither group showed a statistically significantly decrease over baseline.
   “By per-protocol analysis, post-meal walking and basal-plus groups significantly decreased HbA1c by 0.13(range:-0.74 to 1.08) and 0.26(range:-0.8 to 0.08) %, respectively.”

**Answer:** This statement has actually been corrected in the first revised manuscript but may have been missed. The statement that has already been in the Abstract is “There was no significant differences in HbA1c reduction from baseline in each group and between groups in both intention-to-treat and per-protocol analysis”

2. Line 145: Indicate if the alpha level was one- or two-sided. The beta for a power of 0.80 is 0.20. Please clarify.
3. Line 146: This statement is technically incorrect. “At least 14 participants were needed to demonstrate the statistical significance.” Possibly something to this effect would be more appropriate, “A paired-tests with an alternative hypothesis mean of 0.5 and a standard deviation of 0.65 requires a sample size of 14 to attain 80% power, assuming a two-sided alpha of 0.05.” Possibly this test should be one-sided since a decrease in HbA1c is expected.

**Answer:** Those statements have been corrected and revised according to the reviewer’s suggestion.

4. Line 149: Indicate the underlying covariance structure used in the linear mixed models and the criteria for choosing it.

**Answer:**  We explore data in each patient and it showed random intercept and random slope. So we decided to choose random coefficient model for our outcome analysis.

5. Line 189-191: Provide the overall p-value for comparing times 0, 3 and 6. If the overall p-value is significant use a multiple comparison tests to summarize pairwise differences.

**Answer:** We thank the reviewer for this suggestion. Actually there was no overall change of fructosamine levels after intervention, therefore the result of fructosamine measurements has been revised in the Result and Discussion sections. Figure of fructosamine results has also been revised accordingly.

6. Both Figures 1 and 2 display nearly identical data. Include only one of these.

**Answer:** Fig 2 has been removed and replaced with Fig 3.
